# Reciprocal Associations between Depressive Symptoms and Mastery among Older Adults; Black-White Differences

**DOI:** 10.3389/fnagi.2016.00279

**Published:** 2017-01-05

**Authors:** Shervin Assari, Maryam M. Lankarani

**Affiliations:** ^1^Department of Psychiatry, University of MichiganAnn Arbor, MI, USA; ^2^Center for Research on Ethnicity, Culture and Health, School of Public Health, University of MichiganAnn Arbor, MI, USA; ^3^Medicine and Health Promotion InstituteTehran, Iran

**Keywords:** population groups, ethnic groups, african americans, depressive symptoms, depression, mastery, self-efficacy

## Abstract

**Purpose:** Although higher levels of depressive symptoms and lower levels of sense of mastery tend to be comorbid, limited information exists on racial differences in the longitudinal associations between the two over time. The current study compared Black and White American older adults for the longitudinal links between depressive symptoms and mastery in the United States.

**Methods:** Using data from the Religion, Aging, and Health Survey, 2001–2004, this longitudinal cohort study followed 1493 Black (*n* = 734) and White (*n* = 759) elderly individuals (age 66 or more) for 3 years. Depressive symptoms [Center for Epidemiological Studies-Depression scale (CES-D), 8 items] and mastery (Pearlin Mastery Scale, 7 items) were measured in 2001 and 2004. Demographics, socio-economics, and physical health were covariates and race was the focal moderator. Multi-group structural equation modeling was used for data analysis, where groups were defined based on race.

**Results:** Among White but not Black older adults, higher levels of depressive symptoms at baseline predicted a greater decline in sense of mastery over 3 years of follow-up. Similarly among Whites but not Blacks, individuals with lower mastery at baseline developed more depressive symptoms over time.

**Conclusion:** Findings are indicative of Black-White differences in reciprocal associations between depressive symptoms and mastery over time. Race alters how depression is linked to changes in evaluation of self (e.g., mastery) over time.

## Introduction

According to the Black-White health paradox, one of the mysteries of health research (Keyes, 2009; Barnes et al., 2013; Mouzon, 2013, 2014; Assari et al., 2015; Watkins et al., 2015), despite higher exposure to psychosocial and economic stress and adversities, Blacks less frequently meet criteria for depression compared to Whites (Owen, 1996; Lindhorst et al., 2007; Signorello et al., 2007; Williams et al., 2007; González et al., 2010; Cabassa et al., 2013; Jackson et al., 2013; Johnson-Lawrence et al., 2013). In line with this paradox, Blacks and Whites differ in psychosocial and health factors that correlate with depression and depressive symptoms (Sachs-Ericsson et al., 2007; Gavin et al., 2010; Lewis et al., 2011; Barnes et al., 2013; Capistrant et al., 2013; Assari and Lankarani, 2014; Assari, 2014d; Assari et al., 2016b). This hypothesis is supported by research showing that depression increases the risk of chronic medical conditions among Whites but not Blacks (Assari et al., 2015, 2016a). Depressive symptoms predict chronic medical conditions (Assari et al., 2015) and all-cause (Assari et al., 2016a; Moazen-Zadeh and Assari, 2016) and cause-specific (Assari and Burgard, 2015) mortality among Whites but not Blacks. In another set of studies, depressive symptoms have failed to correlate with expected biological markers, such as inflammatory markers (Case, 2014; Stewart, 2016; Vrany et al., 2016).

The first possible explanation proposed by Keyes for the Black-White health paradox is that Blacks may experience some levels of growth or flourishing in the presence of adversities (Keyes, 2009). Assari has found that Blacks are systematically more resilient to the effects of psycho-social factors, which may reflect an adaptive resilience of Blacks as a result of life under economic and social adversity, or culture (Krause, 2002; Assari et al., 2016c; Assari, 2016a,b,c; Assari and Lankarani, 2016b; Assari and Dejman, 2016). These two explanations are supported by studies documenting higher availability and efficacy of interpersonal psychosocial resources, such as social support and religion, for Blacks than Whites (Lincoln et al., 2003; Keyes, 2009; Barnes et al., 2013; Mouzon, 2013, 2014; Assari et al., 2015). Jackson has suggested that Blacks use behavioral coping mechanisms that lower the risk of emotional problems, however, increase the risk of metabolic and cardiovascular conditions (Jackson and Knight, 2006; Mezuk et al., 2010, 2013).

There are also a series of methodological explanations that attribute this paradox to measurement errors or selection biases. For instance, some evidence suggests that depression measures that are designed and validated for Whites fail to capture depression in Blacks (Moazen-Zadeh and Assari, 2016). In one study, baseline depressive symptoms were predictive of the subsequent risk of Major Depressive Disorder (MDD) after 15 years among Whites but not Blacks (Moazen-Zadeh and Assari, 2016). This view is also supported by the literature suggesting lower validity of depressive measures among Blacks (Williams et al., 2007) as well as studies that depression may differently present among Blacks and Whites (Brown et al., 1996; Blazer et al., 1998; Zayas et al., 2002).

The third generation of explanations recently proposed by Assari is that depression differently influences dysfunctional attitudes about self (e.g., mastery), others, and the future (e.g., Hope) (Assari, 2016a; Assari and Lankarani, 2016a; Assari and Dejman, 2016). In this view, depression is qualitatively different among Blacks and Whites; thus Blacks and Whites may differ in the link between depression and mastery, defined as a sense of having control over the forces that affect one's life (Fok et al., 2012). We argue that high levels of psychosocial resources, such as social support and religion, may make Blacks more resistant to decline in mastery in the face of social adversities (Lincoln et al., 2003; Assari, 2013; Mouzon, 2013, 2014). Level of mastery will have implications on how stress may affect depression among Blacks and Whites (Assari and Lankarani, 2016c). This view is also supported by studies suggesting that locus of control, mastery, self-efficacy, and control over life may not have similar meanings in Whites and Blacks (Deaton and Lubotsky, 2003; Dressler et al., 2005; Dowd and Zajacova, 2007; Assari, 2014c, 2016e; Assari et al., 2016c). In a number of studies, Assari has shown that depression differently influences dysfunctional attitudes about self, others, and the future (Assari, 2016a; Assari and Lankarani, 2016a; Assari and Dejman, 2016). For instance, lower levels of these control beliefs may have more significant health consequences for Whites than Blacks (Assari, 2016c).

Built on the *differential effect hypothesis* (Assari, 2016b,d; Assari and Lankarani, 2016b; Assari and Sonnega, 2016), we conducted the current study to compare Black and White American older adults for the reciprocal longitudinal associations between depressive symptoms and mastery over time. This hypothesis is in line with what Belsky and others have called ‘differential susceptibility to environmental influences’ or ‘differential susceptibility to the context’ (Belsky, 1997; Boyce and Ellis, 2005; Belsky et al., 2007; Belsky and Pluess, 2009). Given that previous studies have shown that religion and social support may be more available and effective for Blacks compared to Whites (Krause, 2006a; Skarupski et al., 2013) paired with the finding that Blacks are more resilient to the effect of psychosocial factors (Krause, 2002; Assari, 2016a,b,c; Assari and Lankarani, 2016b; Assari and Dejman, 2016; Assari et al., 2016c), we expected to observe weaker reciprocal longitudinal links between depressive symptoms and mastery among Blacks compared to Whites. We used nationally representative data to generate results that are generalizable to the U.S. population of older adults.

## Methods

### Design and setting

With a longitudinal panel design, data came from Waves 1 and 2 of the Religion, Aging, and Health Survey, 2001–2004. The study is a 3 year follow up of a nationally representative household sample of Black and White older adults in the U.S. (Krause, 2006b, 2009).

### Sampling and participants

The study participants were either White or Black older adults. Older Blacks were over-sampled in the survey. All participants were non-institutionalized English speaking people of ages greater than 65 years. The study population was limited to those who were either Christians or those who were never associated with any faith.

### Measures

Race, demographic data (age and gender), and socio economic status (education) were measured at baseline in 2001. Number of chronic medical conditions (13 chronic medical conditions) was measured in 2004. Depressive symptoms and mastery were measured in 2001 and 2004.

#### Depressive symptoms

An 8–item Center for Epidemiological Studies-Depression scale (CES-D) (Radloff, 1977) was used to measure depressive symptoms in 2001 and 2004. Items measured the extent to which respondents felt depressed or had somatic symptoms. Abbreviated CES-D measures have shown acceptable reliability and similar validity as compared to the original 20–item version (Andresen et al., 1994; Zhang et al., 2012; Amtmann et al., 2014). Items used were as the following: (1) I felt I could not shake off the blues even with the help of my family and friends, (2) I felt depressed, (3) I had crying spells, (4) I felt sad, (5) I did not feel like eating, my appetite was poor, (6) I felt that everything I did was an effort, (7) My sleep was restless, and (8) I could not get going. All these items were selected from the negative domain of the CES-D; positive affect and interpersonal items were not reflected in this version of the CES-D. Item responses were 1 (“rarely or none”) to 4 (“most or all of the time”). We calculated the mean score which treated depressive symptoms as a continuous measure, with a potential range from 1 to 4. Higher scores indicated more severe depressive symptoms. (Abu-Raiya et al., 2016; Hayward and Krause, 2016)

#### Mastery

We used seven items from the Pearlin Mastery Scale (Cairney and Krause, 2008). Items included (1) You have little control over the things that happen to you, (2) There is really no way you can solve some of the problems you have, (3) There is little you can do to change many of the important things in your life, (4) Sometimes you feel that you are being pushed around in life, (5) What happens to you in the future mostly depends upon you, (6) You can do just about anything you really set your mind to, and (7) You often feel helpless in dealing with the problems of life (Pearlin and Pioli, 2003). This construct is similar to sense of control (Wheaton, 1983; Rodin, 1986; Mirowsky, 1995, 1997). We calculated a mean score where a higher score was indicative of a greater sense of mastery (range = 0–4). Some of the items were reverse coded. The internal reliability of the scale (Cronbach's alpha) at Wave 1 was 0.94.

#### Number of chronic medical conditions

The presence of the following chronic medical conditions were measured during the past 12 months: (1) hypertension, (2) heart problem, (3) diabetes, (4) cancer, (5) kidney disease, (6) arthritis or rheumatism, (7) intestinal disorders, (8) liver disease, (9) urinary tract disorders, (10) eye diseases, (11) any respiratory disease, and (12) any other major health problem. Possible responses included yes [1], no [0], and do not know (missing data). Number of conditions potentially ranged between 0 and 12, where a higher score was indicative of more chronic conditions. (Watkins et al., 2015) We decided to control for chronic medical conditions as physical health is correlated with mastery and depressive symptoms and may confound their link. Medical conditions may, however, be differently linked to depression based on race (MacKinnon et al., 2000; Lynch et al., 2010; Lewis et al., 2011; Barnes et al., 2013; Capistrant et al., 2013; Assari and Burgard, 2015; Assari et al., 2015).

### Statistical analysis

We used SPSS 20.0 (IBM Corp, Armonk, NY) for univariate and bivariate analyses. We used AMOS 20 (IBM Corp, Armonk, NY) for multivariable analysis. For bivariate associations, Pearson's correlations tests, independent sample *t*-tests, and paired *t*-tests were used. For multivariable analysis, we ran multi-group structural equation modeling (SEM) to test if baseline depressive symptoms and mastery predict subsequent depressive symptoms and mastery, while age, education, gender, and chronic medical conditions were controlled. We ran a multi-group model where groups were defined based on race (Kline, 2010). *P* < 0.05 was considered significant.

The Amos software computes maximum likelihood estimates in the presence of missing data (Allison, 2002; Arbuckle, 2012). Model fit was evaluated by examining the chi-square statistic, the comparative fit index (CFI), and the root mean square error of approximation (RMSEA). A non-significant chi-square statistic, a chi-square to degrees of freedom ratio of less than 4, a CFI above 0.95, and a RMSEA value of 0.06 or less were considered as indicators of good fit (Hu and Bentler, 1999; Lei and Lomax, 2005).

## Results

### Descriptive statistics

The study followed 1493 older adults (age 65 or greater) for 3 years. This sample included 734 Blacks and 759 Whites. Descriptive statistics overall and also based on race are shown in Table 1. While age was not significantly different between the racial groups, Blacks were more female, and had lower education. Blacks reported more depressive symptoms than Whites.

**Table 1:** **Descriptive Statistics for the analytic sample, stratified by race and overall**.

	**OR**	**95% CI**		**P**	**OR**	**95% CI**
	**All**		**Whites**		**Blacks**	
	**Mean n**	**SD %**	**Mean n**	**SD %**	**Mean n**	**SD %**
GENDER^*^						
Male	570	38.2	314	41.4	256	34.9
Female	923	61.8	445	58.6	478	65.1
**EDUCATION**						
High school diploma or higher	872	59.0	552	73.4	320	44.0
Less than High school diploma	607	41.0	200	26.6	407	56.0
	Mean	SD	Mean	SD	Mean	SD
Age	75.14	6.66	75.37	6.82	74.91	6.49
Medical conditions	1.77	1.82	1.74	1.81	1.78	1.83
Depressive symptoms ^*^	1.49	0.69	1.47	0.62	1.52	0.77
Mastery						

* *p < 0.05*.

### Multivariable model

**Figure 1:**
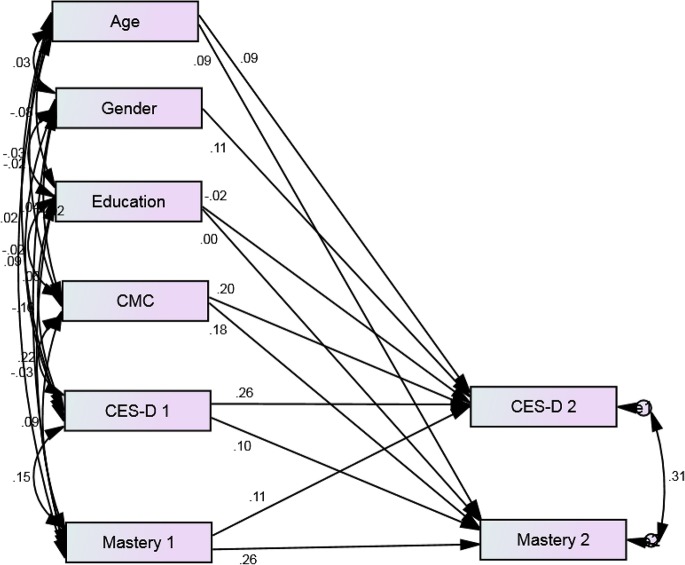
**Standardized regression weights for longitudinal associations between depressive symptoms and mastery over 3 years among White older adults from 2001 to 2004**. *P* = 0.468, CMIN = 1.519, DF = 2, CMIN/DF = 0.759, CFI = 1.000, RMSEA = 0.000, 90% CI = 0 000–0.057.

**Figure 2:**
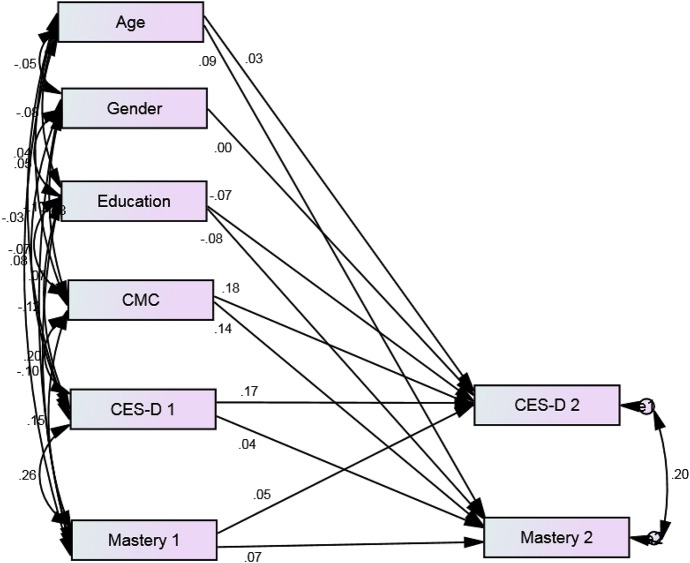
**Standardized regression weights for longitudinal associations between depressive symptoms and mastery over 3 years among Black older adults from 2001 to 2004**. *P* = 0.468, CMIN = 1.519, DF = 2, CMIN/DF = 0.759, CFI = 1.000, RMSEA = 0.000, 90% CI = 0.000–0.057.

Our model showed an excellent fit to the data (*p* = 0.468, CMIN = 1.519, DF = 2, CMIN/DF = 0.759, CFI = 1.000, RMSEA = 0.000, 90% CI = 0.000–0.057; Figures 1, 2). Depressive symptoms at baseline was associated with decline in mastery over time among Whites (β = 0.10, *p* = 0.013) but not Blacks (β = 0.04, *p* = 0.369). Mastery at baseline was also predictive of an increase in depressive symptoms over time among Whites (β = 0.11, *p* = 0.007) but not Blacks (β = 0.05, *p* = 0.255). Baseline age and gender were associated with change in depressive symptoms for Whites but not Blacks. Number of medical conditions was predictive of change in mastery and depressive symptoms among Whites and Blacks (Table 2, Figures 1, 2).

**Table 2:** **Longitudinal associations between depressive symptoms and mastery over 3 years among White and Black older adults**.

			**Whites**	**Blacks**		
			**B (SE)**	***P***	**B**	***P***
W1 Depressive symptoms	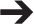	W2 Depressive symptoms	0.26(0.05)	<0.001	0.17(0.06)	<0.001
W1 Mastery	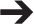	W2 Mastery	0.26(0.05)	<0.001	0.07(0.05)	0.115
W1 Mastery	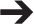	W2 Depressive symptoms	0.11(0.05)	0.007	0.05(0.07)	0.255
W1 Depressive symptoms	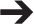	W2 Mastery	0.10(0.04)	0.013	0.04(0.04)	0.369
W1 Age	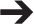	W2 Depressive symptoms	0.09(0.00)	0.027	0.03(0.01)	0.509
W1 Age	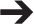	W2 Mastery	0.09(0.00)	0.025	0.09(0.00)	0.048
W1 Gender (Female)	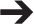	W2 Depressive symptoms	0.11(0.05)	0.003	0.00(0.07)	0.942
W2 CMC	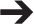	W2 Depressive symptoms	0.20(0.02)	<0.001	0.18(0.02)	<0.001
W2 CMC	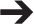	W2 Mastery	0.18(0.01)	<0.001	0.14(0.02)	0.003
W1 Education (High school diploma)	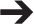	W2 Mastery	0.00(0.05)	0.955	−0.08(0.05)	0.093
W1 Education (High school diploma)	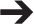	W2 Depressive symptoms	−0.02(0.06)	0.648	−0.07(0.07)	0.142

*P = 0.468, CMIN = 1.519, DF = 2, CMIN/DF = 0.759, CFI = 1.000, RMSEA = 0.000, 90% CI = 0.000–0.057*.

## Discussion

In a nationally representative sample of older adults in U.S., we found that sense of mastery and depressive symptoms have reciprocal links among Whites but not Blacks. These Black-White differences are in line with other racial differences in psychosocial and medical correlates of depression (Sachs-Ericsson et al., 2007; Gavin et al., 2010; Lewis et al., 2011; Capistrant et al., 2013; Assari and Lankarani, 2014; Assari, 2014d; Assari and Burgard, 2015; Assari et al., 2015, 2016a,b,c; Watkins et al., 2015; Moazen-Zadeh and Assari, 2016) that collectively support the differential effect hypothesis and differential susceptibility to context.

These findings enhance our understanding regarding the relevance of Beck's negative cognitive triad to racially diverse groups. Based on Beck's theory, depression is a dysfunctional evaluation of self, others, and the future (Beck et al., 1990). It has been previously shown that depression and hopelessness (dysfunctional evaluation of the future) have stronger links among Whites than Blacks (Assari and Lankarani, 2016a). These findings suggest that race may alter how distorted evaluation of self, others, and the future reflects depression among populations. These findings have major clinical implications for psychotherapy and cognitive therapy of depression in diverse populations (Assari and Lankarani, 2016a).

Lincoln (Lincoln et al., 2003) and others (Assari, 2013; Assari, under review) have argued that social and psychological factors operate differently across racial and ethnic groups. Psychosocial processes may operate in unique manners that are distinct across each racial and ethnic group (Lincoln et al., 2003). In this view, the assumption of similarity between Blacks and Whites is the result of an absence of previous studies on race/ethnic differences, and does not have empirical evidence (Hunt, 1996; Hunt et al., 2000; Lincoln et al., 2003; McDowell, 2010; Assari, 2016c). Our findings suggest that the psychosocial processes that result in depression may depend on race.

Racial groups have unique histories, values, and cultures, which combine with life circumstances and experiences that make them differently vulnerable or resilient to risk and protective factors. In this view, the very same social factors, and the very same psychosocial theory may differently operate across all social groups (Lincoln et al., 2003). Understanding of group differences in psychosocial processes that contribute to health and illness is essential for the promotion of health and wellbeing through altering social or psychological factors across diverse populations (Lincoln et al., 2003). This argument is core to the *differential effect hypothesis* (Assari, 2016d).

It has been shown that overall social support and support from fellow church members are stronger predictive factors against psychological distress and depressive symptoms among Blacks compared to Whites (Lincoln et al., 2003; Krause, 2006a; Assari and Burgard, 2015; Assari, under review). In 2013, Assari showed that religious (church-based) social support fully mediated the association between church attendance and overall life satisfaction for Blacks but not Whites (Assari, 2013). These are all in line with what Skarupski et al., called the Blacks' “faith advantage in health” (Skarupski et al., 2013).

Our finding adds to the existing knowledge on racial differences in the complex associations between socioeconomic status (SES), psychosocial resources (e.g., social support, coping, stress), depression, and health (Drevenstedt, 1998; Krause, 2002; Sachs-Ericsson et al., 2007; Cheng et al., 2010; Gariepy et al., 2010; Gavin et al., 2010; Cohen et al., 2012; Flegal et al., 2013; Assari, 2014a,b; Patel et al., 2014; Assari et al., 2016c). Race modifies how SES affects depression, health behaviors, and mortality (Flegal et al., 2013; Patel et al., 2014) and how depression is linked to obesity (Sachs-Ericsson et al., 2007; Gavin et al., 2010; Assari, 2014a,b), chronic medical conditions (Assari and Lankarani, 2014; Watkins et al., 2015), self-rated health (Assari, 2014d; Assari and Burgard, 2015; Assari et al., 2015), and mortality (Assari and Burgard, 2015; Assari et al., 2016a).

Race differences in vulnerabilities to the effect of risk and protective factors may be a consequence of racial differences in exposure to psychosocial risk and protective factors (Ferraro and Kelley-Moore, 2001; Lee et al., 2007; Assari and Burgard, 2015; Assari et al., 2016c), race differences in the nature of depression or mastery (Moazen-Zadeh and Assari, 2016), or racial differences in what psychosocial constructs reflect (Assari et al., 2016c). These non-specific racial differences suggest that race does not have a direct effect on health, or simply through SES, but has contextual effects that alter how resources and risk factors impact physical or mental health (Capistrant et al., 2013; Assari, 2014a,c,d, 2015; Assari et al., 2015, 2016c).

The missing link between depressive symptoms and mastery among Blacks may explain why depressive symptoms have weaker effects on self-rated health, medical conditions, and mortality among Blacks compared to Whites (Drevenstedt, 1998; Ferraro and Kelley-Moore, 2001; Dowd and Zajacova, 2007; Lee et al., 2007; Assari and Burgard, 2015; Assari et al., 2016d). In a consistent pattern, regardless of the type of the predictor, psychological variables better predict physical and mental health outcomes for Whites compared to Blacks (Ferraro and Kelley-Moore, 2001; Dowd and Zajacova, 2007; Lee et al., 2007; Assari, 2016e). These suggest higher resilience of Blacks toward different risk factors, possibly due to historical exposure to adversity.

Our findings may have implications for the elimination of racial health disparities in the US, which have existed for several decades (Deaton and Lubotsky, 2003; Dressler et al., 2005). This finding may also be relevant to the ongoing increasing trends of mortality due to mental disorders, depression, and alcohol use among White men (Case and Deaton, 2015). We believe that mastery is central to trajectories of mental disorders and chronic disease, particularly for Whites.

Our study is subject to a number of limitations. First and foremost, we measured depressive symptoms, not major depressive disorder, which would have required a structural interview. We also did not measure history of anti depressant use, which affects the course of depression. Third, we did not control for potential confounders, such as stress, religiosity, social support, and cognitive ability. Fourth, we used short versions of standard measures of mastery and depressive symptoms. Despite these limitations, this study was one of the firsts on racial differences in the links between mastery and depressive symptoms over time among older adults. Using a nationally representative sample and large sample size of Blacks can be listed as strengths of this study.

To conclude, race alters the reciprocal and longitudinal associations between depressive symptoms and sense of mastery over time. Black-White differences in the link between depression and mastery may explain why depression and depressive symptoms better predict chronic medical conditions and mortality among Whites than Blacks. Future research should test whether racial differences in the paths between depression and mastery is due to racial differences in quality of depression and control beliefs, racial differences in availability and effects of psychosocial factors (e.g., SES, religiosity, and social support) or differential salience of dysfunctional attitudes about self, others, and the future explain these differences between Blacks and Whites.

## Ethics statement

All procedures followed were in accordance with the ethical standards of the responsible committee on human experimentation (institutional and national) with the Helsinki Declaration of 1975, as revised in 2000. Informed consent was obtained from all participants included in the study. The University of Michigan Institutional review board (IRB) approved the study protocol.

## Author contributions

SA designed and analyzed this work, and contributed to revision. ML drafted and revised the paper. Both authors confirmed the last version.

## Funding

The Religion, Aging, and Health Survey was supported by National Institute on Aging (PI: Neal Krause R01 AG014749), and per the NIH Public Access Policy requires that peer-reviewed research publications generated with NIH support are made available to the public through PubMed Central. NIH is not responsible for the data collection or analyses represented in this article. Data was accessed through The Interuniversity Consortium for Political and Social Research (ICPSR), the Institute of Social Research, University of Michigan.

### Conflict of interest statement

The authors declare that the research was conducted in the absence of any commercial or financial relationships that could be construed as a potential conflict of interest.
